# Editorial: Gut microbiota-derived metabolites and cardiovascular diseases

**DOI:** 10.3389/fcimb.2025.1661489

**Published:** 2025-08-29

**Authors:** Xiao Cui, Jing Li, Tao Yang

**Affiliations:** ^1^ Department of Cardiology, The First Affiliated Hospital, Zhejiang University School of Medicine, Hangzhou, China; ^2^ Medical Research Center, Beijing Institute of Respiratory Medicine and Beijing Chaoyang Hospital, Capital Medical University, Beijing, China; ^3^ Department of Cardiology, Beijing Chaoyang Hospital, Capital Medical University, Beijing, China; ^4^ Center for Hypertension and Precision Medicine, Microbiome Consortium, Department of Physiology and Pharmacology, University of Toledo College of Medicine and Life Sciences, Toledo, OH, United States

**Keywords:** gut microbiota, cardiovascular diseases, hyperlipidemia (HL), heart failure with preserved ejection fraction (HFpEF), fecal microbiota transplantation

Cardiovascular diseases are the leading cause of death worldwide and a huge burden on global health and economies. These diseases primarily impact the heart and blood vessels, and include coronary artery disease, heart failure, hypertension, and aortic aneurysms (C. Global Cardiovascular Risk et al., 2023). Although Cardiovascular diseases are treatable in clinical settings, significant difficulties remain. Late diagnosis, chronic nature, and medication resistance all impede effective management (C. Global Cardiovascular Risk et al., 2023). Furthermore, the common coexistence of cardiovascular diseases and metabolic illnesses needs a more complex treatment regimen, which makes patient care even more complicated.

Gut microbes residing in the gastrointestinal tract not only play a vital role in energy acquisition by metabolizing dietary nutrients but also serve as essential regulators of human physiological functions, particularly metabolic homeostasis (Gabriel and Ferguson, 2023). They are crucial in the development of various diseases (Xavier, 2016). Under normal physiological conditions, the interactions between the gut microbiota and the host are governed by mechanisms such as immune tolerance and autonomic regulation. However, an imbalance in the gut microbiota can disrupt host homeostasis, leading to immune overactivation, autonomic dysregulation, and devastating changes in the metabolism of both gut microbiota and the host (Hou et al., 2022; Gabriel and Ferguson, 2023). As research in this field expands, our Research Topic is organized to include cutting-edge findings, offering both positive and negative data on the relationship between gut microbiota and cardiovascular diseases.

Hyperlipidemia and diabetes are common complications associated with cardiovascular diseases. Both are considered metabolic syndromes, characterized by disturbed metabolism in lipid and glucose, respectively. In a study, Han et al. utilized integrative metagenomic and metabolomic analyses to investigate the possible association between glycerolipid metabolism and gut microbiome in the development of high fat diet (HFD) induced hyperlipidemia. This study was performed using a hamster model, which exhibits pathophysiological characteristics more closely aligned with those of humans than mice. *Faecalibaculum* and *Allobaculum* are ound increased in HFD, associated with higher levels of monoacylglycerols (18:2), suggesting the role of both gut microbes in hyperlipidemia. In a review, Xu et al. summarized current knowledge on imidazole propionate (ImP) and proposed its potential involvement in type 2 diabetes and cardiovascular diseases. An increasing number of gut microbiota-derived metabolites have been implicated in cardiovascular diseases pathogenesis (Yang et al., 2021). Among these, bile acids are further modified (i.e. conjugation, dehydroxylation) by gut bacteria in the intestine. Bile acids are potent metabolic and immune signaling molecules synthesized from cholesterol in the liver. Disruption of bile acid signaling, due to microbial dysbiosis or impaired host–microbe interactions, has been associated with the development and progression of cardiovascular diseases, including hypertension (Chakraborty et al., 2023) and other metabolic disorders (Cai et al., 2022). Additionally, short-chain fatty acids (SCFAs), produced as fermentation byproducts of prebiotic fibers, exhibit immunomodulatory effects and confer several cardiovascular benefits (Xu et al., 2022).

ImP, a histidine-derived microbial metabolite, is exclusively produced by bacteria possessing the urocanate pathway. Elevated levels of ImP have been found in individuals with low bacterial gene richness and the Bacteroides 2 enterotype (Molinaro et al., 2020). Experimental studies have shown that ImP can induce intestinal inflammation, impair intestinal barrier integrity, reduce goblet cell density, and attenuate the glucose-lowering effect of metformin. Although limited, emerging evidence also suggests a potential link between ImP and cardiovascular health; for instance, ImP levels have been correlated with diastolic blood pressure in obese individuals (van Son et al., 2021). Despite its promise as a therapeutic target, research specifically addressing ImP’s role in cardiovascular diseases remains sparse and warrants further investigation.

Heart failure with preserved ejection fraction (HFpEF), aortic aneurysm, and acute coronary syndrome (ACS) are prevalent forms of cardiac dysfunction under the broader category of cardiovascular diseases. HFpEF, which accounts for more than half of all heart failure cases, is a systemic and multifactorial condition characterized by impaired ventricular relaxation, diastolic dysfunction, and increased myocardial stiffness and fibrosis. It often coexists with metabolic comorbidities. Zhou et al. explored the role of the gut microbiota in HFpEF pathogenesis, noting consistent dysregulation among patients despite population heterogeneity (Huang et al., 2021). Two major contributing mechanisms include gut microbiota-driven immune activation—particularly via the NLRP3 inflammasome–IL-1/IL-6 axis—and altered microbial metabolites such as trimethylamine-N-oxide (TMAO), short-chain fatty acids (SCFAs), and bile acids.

In another study, Sun et al. investigated the association between gut microbiota and aortic aneurysm diseases using bidirectional two-sample Mendelian randomization (MR), drawing on large-scale datasets such as MiBioGen, the Dutch Microbiome Project, FinnGen, UK Biobank, and the Michigan Genomics Initiative. While preliminary associations were observed, none remained statistically significant after correcting for false discovery rate (FDR). MR’s use of genetic variants as instrumental variables helps mitigate confounding, yet the complex etiology of aortic aneurysms likely involves multiple non-microbiota-related mechanisms. Additionally, Fang et al. assessed the link between Helicobacter pylori infection and ACS risk in 280 Chinese participants. Their findings revealed that although H. pylori did not significantly alter lipid metabolism, it increased ACS risk by fourfold, indicating alternative pathogenic pathways unrelated to lipid regulation.

Fecal microbiota transplantation (FMT) is a powerful experimental approach used to investigate the role of gut microbiota in health and disease (Hou et al., 2022). By transplanting fecal samples from donors into recipients that have been pre-treated with broad-spectrum antibiotics to deplete their native gut microbiota, researchers can assess the impact of donor-derived microbial communities by observing physiological and pathological outcomes in the recipients. In two separate studies, Cai et al. demonstrated that gut microbiota alterations induced by sleep deprivation were responsible for cardiac dysfunction, while Fan et al. reviewed evidence showing that the benefits of Qian Yang Yu Yin granules in improving organ damage were partially mediated through modulation of the gut microbiota. Qian Yang Yu Yin granules, a traditional Chinese herbal formulation, are rooted in the principles of Traditional Chinese Medicine—particularly the concept of restoring balance between Yin and Yang. The name “Qian Yang Yu Yin” reflects this philosophy, meaning to suppress excess Yang and nourish Yin to reestablish internal harmony. The granules of Qian Yang Yu Yin have demonstrated therapeutic benefits in renal injury (Qian et al., 2021) and hypertensive cardiac remodeling (Xu et al., 2025). Therefore, these findings highlight the potential of microbiota-targeted interventions—including Traditional Chinese Medicine—as promising therapeutic strategies for hypertension and cardiovascular complications.

The role of gut microbiota in cardiovascular diseases is an increasingly active and promising area of research. Given that gut microbial composition is influenced by a variety of environmental and host genetic factors, studies in this field must carefully control for variables such as sex, age, animal strain, diurnal timing, and even bedding materials to ensure reproducibility and accuracy. Moving forward, future research should aim to pinpoint individual microbial species or specific metabolites that drive cardiovascular outcomes, as such focused investigations hold the greatest potential for clinical translation and targeted therapeutic development.
